# Research Progress of ATGs Involved in Plant Immunity and NPR1 Metabolism

**DOI:** 10.3390/ijms222212093

**Published:** 2021-11-09

**Authors:** Shuqin Huang, Baihong Zhang, Wenli Chen

**Affiliations:** 1 MOE Key Laboratory of Laser Life Science, Institute of Laser Life Science, College of Biophotonics, South China Normal University, Guangzhou 510631, China; huangshuqin@m.scnu.edu.cn (S.H.); zhangbyhome@126.com (B.Z.); 2 Guangdong Provincial Key Laboratory of Laser Life Science, College of Biophotonics, South China Normal University, Guangzhou 510631, China; 3 Guangzhou Key Laboratory of Spectral Analysis and Functional Probes, College of Biophotonics, South China Normal University, Guangzhou 510631, China

**Keywords:** *Arabidopsis*, autophagy, NPR1, plant immunity

## Abstract

Autophagy is an important pathway of degrading excess and abnormal proteins and organelles through their engulfment into autophagosomes that subsequently fuse with the vacuole. Autophagy-related genes (ATGs) are essential for the formation of autophagosomes. To date, about 35 ATGs have been identified in *Arabidopsis*, which are involved in the occurrence and regulation of autophagy. Among these, 17 proteins are related to resistance against plant pathogens. The transcription coactivator non-expressor of pathogenesis-related genes 1 (NPR1) is involved in innate immunity and acquired resistance in plants, which regulates most salicylic acid (SA)-responsive genes. This paper mainly summarizes the role of ATGs and NPR1 in plant immunity and the advancement of research on ATGs in NPR1 metabolism, providing a new idea for exploring the relationship between ATGs and NPR1.

## 1. Plant Immunity

### 1.1. PTI and ETI

Plants have evolved a complex immune system to combat the threat from pathogenic microorganisms in nature, including innate and acquired immunity [1,2,3]. It possesses two innate immune defense lines that enable cell-autonomous defense responses upon pathogen infection. For the first line of innate immunity, plant cell surface-localized pattern recognition receptors (PRRs) recognize microbe associated molecular pattern (MAMP) or pathogen-associated molecular pattern (PAMP) to activate pathogen-associated molecular pattern triggered immunity (PAMP-triggered immunity, PTI) [4,5,6]. However, some plant pathogens can produce effectors to inhibit PTI. The other immune defense line is activated by the proteins encoded by resistance genes (*R* genes), these proteins can directly or indirectly recognize the effectors secreted by pathogenic microorganisms. This process is known as effector-triggered immunity (ETI) that usually leads to local programmed cell death (PCD) called hypersensitive response (HR) [7,8]. *R* genes are highly expressed during pathogen infection, most of them encode the nucleotide-binding (NB) domain and Leu-rich repeat (LRR)-containing (NLR) proteins that recognize pathogen effectors and activate ETI, which usually leads to the accumulation of reactive oxygen species (ROS) and HR. Based on the N-terminal structures, NLR proteins can be classified into two categories. TIR-NLR (TNLs) contain the toll/interlcukin-1-reccptor (TIR) region and CC-NLR (CNLs) contain coiled coil (CC) domain [9,10,11,12,13,14,15].

The latest studies have clarified the new mechanism of crosstalk and cooperation between PTI and ETI, they activate many pathways that are closely related to each other and activate plant immune signaling pathways [16,17,18]. ETI enhances PTI responses, including ROS production, callose deposition, and upregulation of gene expression [16]. In addition, ETI-induced HR-PCD is enhanced by PTI [16]. More importantly, knocking out of key genes in the PTI pathway inhibits the ETI. In PRRs/co-receptor *Arabidopsis* mutants, *fls2*/*efr*/*cerk1* (*fec*) and *bak1*/*bkk1*/*cerk1* (*bbc*) mutants, ETI induced by *Pst* DC3000 (*avrRpt2*) was severely impaired [17,18]. It indicates that activation of ETI requires PTI involvement, this finding has major implications on future plant immunity studies.

### 1.2. SAR

Plant system acquired resistance (SAR) can be activated by local defense response, which emits chemical signals to alert neighboring cells and tissues and protect the whole organism [19,20,21,22,23]. Thus, it enables the plant to activate defense responses more quickly, strongly, and effectively when subsequently challenged by pathogens. This requires strict and precise regulation of plant hormones, metabolites, and proteins [24,25,26,27,28]. SAR activation is associated with the accumulation of salicylic acid (SA) and the induction of *pathogenesis-related* (*PR*) genes [29,30,31]. Recent studies have shown that pipecolic acid (Pip) and glycerol-3-phosphate (G3P) stimulate each other’s biosynthesis and act together to trigger intracellular SAR and the emission of plant-to-plant (PTP) cues [32,33].

## 2. ATGs Involved in Plant Resistance to Pathogens

Autophagy is an evolutionary conserved intracellular regulatory mechanism, involving the degradation and recycling of intracellular proteins, metabolites, and organelles. One of its main characteristics is the formation of double-membrane vesicles, known as autophagosomes, which engulf a portion of cytoplasm and transport it into vacuoles for degradation [34,35,36,37]. More than 40 known autophagy-related genes (*ATGs*) that strictly regulate this membrane trafficking process have been identified in yeast [38]. In *Arabidopsis*, many genes with sequence similarity to the yeast *ATGs* have been identified. Current information from *Arabidopsis* database TAIR (https://www.arabidopsis.org/, 26 September 2021) and related literature showed that about 35 *ATGs* have been identified. Except for *ATG14/29/31*, other homologous genes of *ATGs* have been found in yeast [39]. The evolutionary process of autophagy is mainly divided into four steps: (1) ATG1-ATG13 complex and target of rapamycin (TOR) jointly induce autophagy. (2) ATG9 and phosphoinositide-3-kinase (PI3Ks) complex containing ATG6, ATG14, vacuolar protein sorting 15 (VPS15), and VPS34, participate in protein sorting and promote vesicle expansion. (3) Two ubiquitin-like conjugation systems, ATG5-ATG12 and ATG8-phosphatidyl ethanolamine (ATG8-PE) systems, induce the formation of autophagosomes. (4) The fusion of mature autophagosomes with the vacuole [35,36,40,41,42,43].

In recent years, great progress has been made in the identification of ATGs and the study of autophagy pathways. Some of these gene knockout mutations revealed the physiological role of autophagy in nutritional stress (nitrogen and carbon deficiency) and senescence [44,45,46]. In addition, more and more studies have shown that autophagy is also involved in plant immune response [47,48,49,50,51]. Autophagy plays a role in promoting and inhibiting pathogens in host–pathogen interactions. Hosts can induce or inhibit plant autophagy during pathogen infection, which is beneficial to resist pathogen invasion [52]. A recent study revealed the interaction between different ATGs and different pathogen effectors. Researchers found that ATG8 interacted with several effectors, while HrpZ1 targeted ATG8 to enhance autophagy levels and increase the virulence of *Pto* DC3000 *hrcC*, HopF3 targeted ATG8 to suppress autophagy. Although the interactions between ATG1, ATG7, ATG12, and several effectors were found in this study, the exact mechanism of these interactions in plant disease resistance is unclear [52]. Some of *ATGs* knockout mutations exhibited enhanced susceptibility to pathogen infection, such as *atg2*, *atg5*, *atg6*, *atg7*, *atg9*, *atg10*, and *atg18* [13,53,54,55,56,57,58,59,60]. While *atg2* mutants displayed less HR-PCD and ATG4, ATG5 inhibited the occurrence of HR-PCD, *ATG6* antisense plants displayed enhanced HR-PCD during pathogen infection [53,54,55,56,57,58,59,61]. A recent study reported that phosphorylation modification of ATG18a suppressed autophagosome formation during pathogen infection, resulting in compromised plant resistance, which provides evidence for the involvement of autophagy in plant immune regulation [62]. Here, we summarize the interaction between bacteria, fungal effectors, and ATGs as well as the role of autophagy in HR-PCD and resistance regulation (Table 1).

## 3. Roles of NPRs in Plant Immunity

### 3.1. The Structure of NPR1

The transcription coactivator non-expressor of pathogenesis-related genes 1 (NPR1) is a key regulatory factor of SAR, which regulates most SA-responsive genes [30,63,64,65,66]. NPR1 contains an N-terminal BTB/POZ (Broad-Compex, Tramtrack, and BricaBrac/POxvirus and Zinc finger) domain, an ankyrin (ANK) repeat domain, a C-terminal transactivation domain, and a nuclear localization sequence [67,68,69]. NPR1 interacts with TGACG motif-binding factor (TGA) through ANK or BTB/POZ domain [70,71,72]. In the absence of SA, the C-terminal transactivation domain of NPR1 interacts with BTB/POZ domain, which inhibits NPR1 transcriptional coactivator function. The binding of SA to NPR1 leads to conformational changes of NPR1, it functions as a coactivator of gene transcription with the release of the C-terminal transactivation domain from the N-terminal autoinhibitory domain [71,73]. A recent study provided a preliminary understanding of the structure–function relationship of NPR proteins. The SA-binding core (SBC) consisting of amino acids 373–516 in the NPR4 C-terminal domain was identified. Arabidopsis NPR4 and NPR1 share 38.1% sequence identity in their SBC region, they share the structural mechanism of SA recognition. In addition, this study also found that conformational changes of NPR4 SBC could be induced by the binding of SA to NPR1 and NPR4 [74].

### 3.2. NPR1 and Innate Immunity

NPR1 is a master regulator of plant resistance to pathogen stress, which confers immunity through multiple transcription factors [75,76,77]. Research over the last 20 years has revealed the potential molecular mechanism of NPR1 in different cell states. Under normal growth conditions, NPR1 is present in the cytoplasm, stabilized by intermolecular disulfide bonds. Infection by pathogens results in the accumulation of SA and NPR1 oligomer-to-monomer reaction through SA-mediated redox changes in the cell, allowing NPR1 to migrate into the nucleus [75,78,79]. NPR1 indirectly activates *PR* gene expression by interacting with TGA in the nucleus and plays an important role in regulating the PRs protein downstream [63,80,81]. The NPR1 in SA perception promotes TGAs transcriptional activity [82]. Recent studies have shown that NPR1 interacts with cyclin-dependent kinase 8 (CDK8) and enhanced disease susceptibility 1 (EDS1) to promote *PR1* expression in the SA signaling pathway [83,84].

A new study found that the formation of SA-induced NPR1 condensates (SINCs) is mediated by conserved cysteine clusters in intrinsic disorder regions (IDRs) of NPR1 protein. SINCs are rich in stress-responsive proteins, including NB-NLR receptors, oxidative and DNA damage-responsive proteins, and ubiquitination-related proteins. In addition, SINCs are required to form functional NPR1-Cullin 3 RING E3 ligase (CRL3) complex in the cytoplasm. NPR1-CRL3 complex can ubiquitinate and degrade EDS1 and some important ETI regulatory factors such as WRKY transcription factors, thereby promoting cell survival in ETI [85].

### 3.3. NPR3/NPR4 and Plant Immunity

In *Arabidopsis*, the *NPR* family consists of *NPR1* and five *NPR1-like* genes, named *NPR1-like 2* (*NPR2*), *NPR3*, *NPR4*, *BLADE-ON-PETIOLE2* (*BOP2*; *NPR5*), and *BOP1* (*NPR6*) [86,87,88,89]. Each member of the *NPR* family contains a set of highly conserved cysteine residues that are thought to be involved in redox control [30]. It was confirmed that NPR1 and NPR3/NPR4 bind to SA and function as SA receptors, with NPR1 (K_d_ = 223.1 ± 38.85 nM) and NPR3 (K_d_ = 176.7 ± 28.31 nM) binding to SA with similar affinity. However, the affinity of NPR4 (K_d_ = 23.54 ± 2.743 nM) with SA is much higher [82]. Under normal conditions, NPR4 is a ligand of CRL3 substrate that can interact with NPR1, allowing proteasome to continuously ubiquitinate and degrade NPR1. At this time point, NPR3/NPR4 inhibits the expression of defense genes, thereby preventing an autoimmune response [90,91,92]. During SAR, as SA levels increase, SA binds to NPR4, induces the dissociation of NPR1 and NPR4, disrupts the NPR4-Cullin3 E3 ligase complex [90,92]. At this time point, the binding of SA to NPR3/NPR4 inhibits their transcriptional activity, while NPR1 in SA perception enhances its transcriptional activation, both of which are helpful in inducing the expression of defense genes [82]. In addition, studies have shown that NPR3 and NPR4 may promote PCD while NPR1 may inhibit PCD through resistance–avirulence (*R-Avr*) gene interaction [91]. Our previous study found that the expression of *ATGs* and the protein concentrations of ATG7 and ATG8a-PE were lower in *npr3/npr4* mutants than in the wild-type. NPR3 and NPR4 may regulate the production of autophagosomes by promoting two ubiquitin-like conjugated systems [91].

## 4. ATGs Participate in the Regulation of NPR1 Metabolism

### 4.1. Proteasome-Mediated NPR1 Degradation

Pathogen infection causes accumulation of SA thus leads to post-translational modification of NPR1, allowing it to enter into the nucleus. NPR1 is recruited to Cullin3 (CUL3) for ubiquitination and subsequent degradation, this process requires phosphorylation of NPR1 at residues Ser11 and Ser15 [31,93,94,95,96]. The ubiquitination of NPR1 is a gradual process. Only when the polyubiquitination of NPR1 is enhanced by ubiquitin conjugation factor E4 (UBE4), it becomes the target of proteasome degradation [95]. Ubiquitin ligase activities are opposed by ubiquitin specific protease (UBP6/7). UBP6/7 are two proteasome-related deubiquitinases (DUBs) that increase NPR1 longevity [95]. In addition to UBP6/7, other DUBs may also play a role in regulating the expression of SA response genes, but their exact function is still unclear.

Some studies have found that the plant hormones abscisic acid (ABA) and SA antagonistically affect the level of NPR1 in cells. ABA promotes NPR1 degradation through the proteasome pathway mediated by the CUL3-NPR3/NPR4 complex, while SA protects NPR1 from ABA-induced degradation through phosphorylation [97,98,99,100]. AvrPtoB has a U-box E3 ubiquitin ligase domain at the C-terminal and shows a weak interaction with NPR1 under uninduced conditions. SA promotes the interaction between AvrPtoB and NPR1, AvrPtoB mediates NPR1 ubiquitination by E3 ligase and mediates NPR1 degradation via the proteasome pathway [101].

### 4.2. Relationship between ATGs and NPR1

Studies have found that NPR1 regulates *ATGs* expression. NPR1 inhibited the mRNA expression of *ATG1*, *ATG6*, and *ATG8a* during the early HR induced by *Psm* ES4326/*AvrRpt2* [61]. SA analog benzothiadiazole (BTH) was confirmed to induce autophagy through the NPR1-dependent signaling pathway, and NPR1, NPR3, and NPR4 are jointly involved in the regulation of autophagosomes [91]. In addition, several studies have shown that NPR1 affects the phenotype of autophagy-deficient mutants. NPR1 could accelerate the senescence or infection-induced accumulation of ubiquitinated proteins and endoplasmic reticulum stress in *atg2* [54]. Yoshimoto et al. found that BTH could induce senescence and cell death in *atg5* mutants but could not induce senescence and cell death in *atg5 npr1* double mutants, indicating that the cell death phenotype in *atg5* mutants depended on NPR1 under SA induction [57]. Our previous study also found that ATG4 promoted NPR1 degradation by inhibiting the consumption of free SA [61]. In recent years, the relationship between ATGs and NPR1 has been gradually revealed (Table 2), but there are still many problems to be solved.

## 5. Conclusions and Future Perspectives

**Figure 1:**
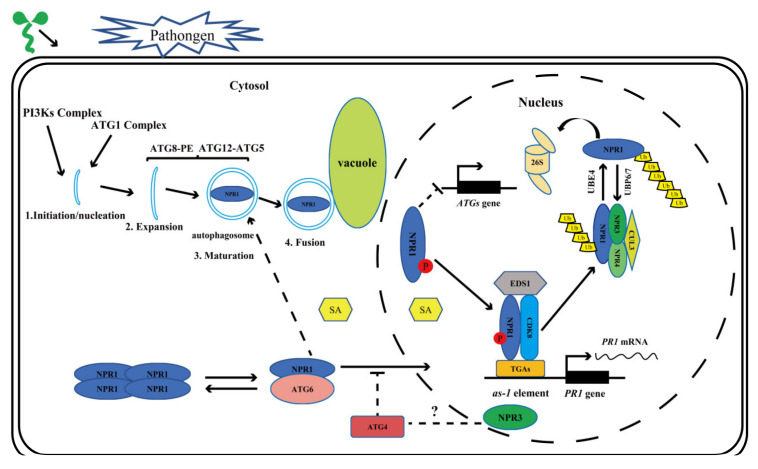
Pattern diagram of autophagy involved in NPR1 regulation in *Arabidopsis*. Autophagy pathway can be divided into four stages: initiation, expansion, maturation, and fusion. Normally, NPR1 exists in the cytoplasm as an oligomer. Upon pathogen infection, SA accumulates in the plant cell. NPR1 was phosphorylated and transferred from cytoplasm to nucleus. In the nucleus, NPR1 forms a protein complex with CDK8 and EDS1, promoting the expression of the *PR1* gene. NPR1 is degraded by the 26S proteasome complex through a series of polyubiquitination processes by CUL3 and UBE4, and its deubiquitination is mediated by UBP6 and UBP7, which are closely linked to 26S proteasome. It is reasonable to speculate that ATG6 may promote the entry of NPR1 into the nucleus, while ATG4 may have the opposite effect.

Autophagy-mediated degradation of proteins and organelles is essential for plant growth, development, maintenance of cell homeostasis, and immune response [34,35,36,37,44,45,46,47,48,49,50,51]. A series of ATGs co-located in the phagophore assembly site (PAS), initiate the process of autophagy. After that, the PI3Ks complex helps to form the nucleation of autophagy, followed by autophagosome membrane elongation [35,36,40,41,42,43,102]. NPR1 activity is regulated by phosphorylation, dephosphorylation, ubiquitination, and deubiquitination, and proteasome is involved in its degradation process (Figure 1). Nevertheless, there are still some questions to be answered, such as whether NPR1, NPR3, and NPR4 have the opposite effects on autophagy regulation and resistance to pathogen invasion? Do they co-repress the production of autophagosomes and the expression of EDS1? In recent years, the role of ATGs (ATG2, ATG5, ATG7, and ATG18a) in plant disease resistance has been gradually revealed (Table 1). In general, the accumulation of SA leads to the outbreak of ROS and further induces autophagy, while autophagy can reduce the production of ROS, thus forming a negative feedback regulation mechanism. ATGs, such as ATG6, can also regulate the occurrence of HR-PCD [48,56,57,103,104]. NPR1 has been proved to inhibit HR-PCD and affect the level of ROS in plants, while it is also affected by the level of ROS [30,91]. Based on this evidence, further research is needed to answer the following questions: Does the mutation or overexpression of *ATGs* affect NPR1 transformation from dimer to monomer? What are the effects of different *ATGs* on NPR1 entering the nucleus? What is the relationship between ATGs and NPR1 regulation of the HR-PCD response? Does autophagy and 26S proteasome co-regulate NPR1 turnover? An in-depth study of these issues will help us to understand how the autophagy pathway participates in the regulation of NPR1 metabolism. A recent study showed that the protein expression of NPR1 was significantly higher in *atg4a4b* than that in wild type under normal condition and the expression of *NPR1* in *atg4a4b* was higher than that in wild type under *avrRpt2* treatment [61]. Based on the above finding and the relationships among ATG6, HR-PCD, and NPR1, a hypothesis regarding ATGs participating in NPR1 metabolism was proposed (Figure 1): ATG6 may promote nuclear translocation of NPR1 by affecting the phosphorylation level of NPR1, while ATG4 may have the opposite effect.

## Figures and Tables

**Table 1:** ATGs (autophagy-related genes) in *Arabidopsis* participate in plant disease resistance.

Gene	Protein	Functions	References
*AT3G61960*	ATG1a	Interacting with AvrRps4-*Pph*, AvrPtoB-*Pto*, HopY1-*Pto*, Rbp001, Rbp002, Rbp005, Urf004, Urf010, Urf012.	[52]
*AT3G19190*	ATG2	*Atg2* mutants displayed enhanced disease resistance to powdery mildew, exhibited enhanced susceptibility upon *D. dadantii* infection. Less HR cell death in *atg2* mutants upon *Pst* DC3000/*avrRpm1* infection.	[53,54,55]
*AT2G44140* *AT3G59950*	ATG4a ATG4b	ATG4 inhibited the occurrence of HR during *Psm* ES4326/*AvrRpt2* infection.	[61]
*AT5G17290*	ATG5	*Atg5* mutants displayed enhanced susceptibility to *Alternaria brassicicola*, *Botrytis cinerea,* and *Plectosphaerella cucumerina.* ATG5 inhibits the growth of *Pst* DC3000 or *Pst* DC3000 containing avirulent factors (*Pst*-*avrB, Pst*-*avrRps4, Pst*-*avrRpm1*) at the early stage of infection, which is necessary to limit PCD induced by *P. syringae*.	[55,57,58,59]
*AT3G61710*	ATG6	*ATG6* antisense plants displayed enhanced HR cell death when infected with virulent *Pst* DC3000 or avirulent *Pst* DC3000/*avrRpm1.*	[56]
*AT5G45900*	ATG7	ATG7 interacts with HrpZ1-*Psy*. *Atg7* mutants displayed enhanced susceptibility to *Alternaria brassicicola*, *Botrytis cinerea,* and avirulent *Pto* DC3000/*AvrRpm1* or *Pto* DC3000/*AvrRps4*.	[13,52,58,59,60]
*AT4G21980*	ATG8a	Interacting with AvrPto, HopF3-*Pph*, HopY1-*Pto*, HrpZ1-*Pph*, Rbp001, Rbp002, Rbp003, Urf003, Urf004. HrpZ1 and HopF3 target ATG8 to enhance and suppress autophagy, respectively.Overexpressing *ATG8a* enhances plant tolerance to *D. dadantii*.	[52,55]
*AT4G04620* *AT2G05630* *AT3G60640* *AT3G06420*	ATG8b ATG8d ATG8g ATG8h	Interacting with HrpZ1. HrpZ1 enhances autophagy levels, increasing the virulence of *Pto* DC3000 *hrcC*.	[52]
*AT4G16520*	ATG8f	Interacting with AvrPtoB-*Pto*, HopF3-*Pph*, HopY1-*Pto*, HrpZ1-*Pph*, Rbp001, Urf004. HrpZ1 and HopF3 target ATG8 to enhance and suppress autophagy, respectively.	[52]
*AT3G15580*	ATG8i	Interacting with AvrB2-*Pph*, AvrB3-*Psy*, AvrPto-*Pto*, HopAQ1-*Pto*, HopO1-2-*Pto*, HopQ1-2-*Pto*, HopX1-*Pto*, HopY1-*Pto*, HrpZ1-*Pph*, HrpZ1-*Psy*, Rbp001, Rbp002, Rbp005, Urf004, Urf012. HrpZ1 enhances autophagy levels, increasing the virulence of *Pto* DC3000 *hrcC*.	[52]
*AT2G31260*	ATG9	*Atg9* mutants displayed enhanced susceptibility to avirulent *Pto* DC3000/*AvrRpm1* or *Pto* DC3000/*AvrRps4*.	[60]
*AT3G07525*	ATG10	Genetic inactivation of *ATG10* resulted in enhanced susceptibility to *Alternaria brassicicola* and *Plectosphaerella cucumerina*, *atg10* mutants showed reduced bacterial growth rates when infected with *Pto* DC3000.	[55,59]
*AT1G54210* *AT3G13970*	ATG12a ATG12b	Interacting with HrpK1-*Pto*, HrpZ1-*Pph*, HrpZ1-*Psy*, Urf003, Urf012.	[52]
*AT3G62770*	ATG18a	*Atg18a* mutants showed enhanced susceptibility to *Alternaria brassicicola*, *Botrytis cinerea,* and showed reduced bacterial growth rates when infected with *Pto* DC3000. Phosphorylation modification of ATG18a suppresses autophagosomes formation during *Botrytis cinerea* infection, which results in compromised plant resistance against *Botrytis cinerea*.	[55,59,62]

**Table 2:** Relationship between ATGs and NPR1 in *Arabidopsis*.

Gene	Protein	Relationship	References
*AT3G61960* AT3G53930	ATG1a ATG1b	NPR1 inhibited the mRNA expression of *ATG1* during *Psm* ES4326/*AvrRpt2* infections.	[61]
*AT3G19190*	ATG2	Accumulation of ubiquitinated proteins and increased ER stress in older *atg2* mutants which were suppressed by mutations in NPR1. NPR1 somehow suppressed cell death in *atg2* mutants upon pathogen infection.	[54]
*AT2G44140* *AT3G59950*	ATG4a ATG4b	ATG4 inhibited the consumption of free SA and alleviated the degradation of NPR1 during *Psm* ES4326*/AvrRpt2* induced autophagy-dependent HR.	[61]
*AT5G17290*	ATG5	Pathogen-induced spread of chlorotic cell death and BTH hypersensitivity in *atg5* mutants required NPR1.	[57]
*AT3G61710*	ATG6	NPR1 inhibited the mRNA expression of *ATG6* during *Psm* ES4326/*AvrRpt2* infections.	[61]
*AT4G21980*	ATG8a	NPR1 inhibited the mRNA expression of *ATG8a* during *Psm* ES4326/*AvrRpt2* infections.	[61]
